# Supplementary figures and images for: A long-term assessment of the multidisciplinary degree of multidisciplinary journals
Source: PLoS One. 2024 Dec 2;19(12):e0314616. doi: 10.1371/journal.pone.0314616 (PMC11611101; doi:10.1371/journal.pone.0314616)

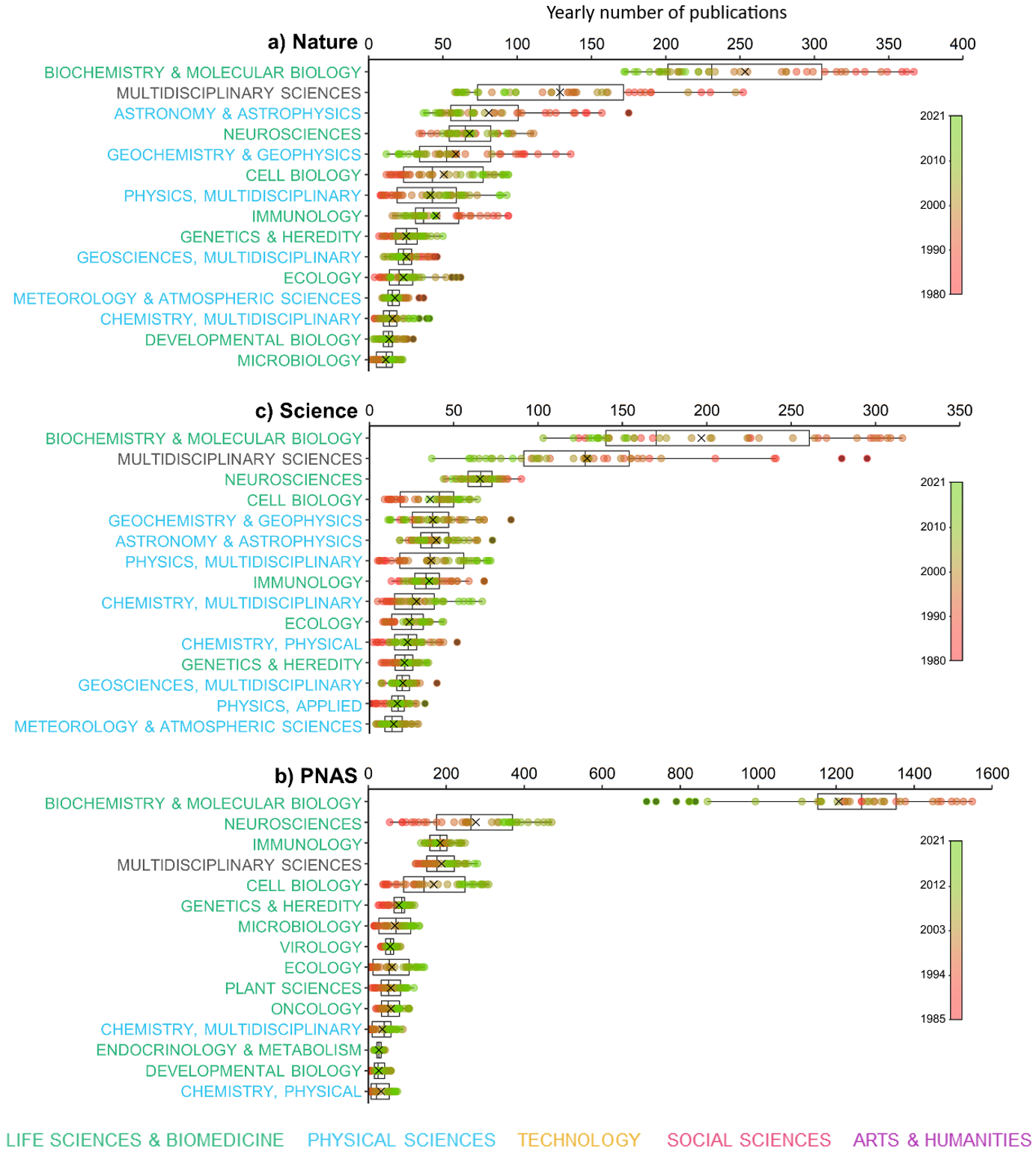

Supplement: S1 Fig — Only the 15 top-ranked research areas are shown, decreasingly sorted by the median. Research areas are classified by branches of knowledge and points are colored according to year. Note that the axes are represented at different scales. (TIF) [file pone.0314616.s001.tif]

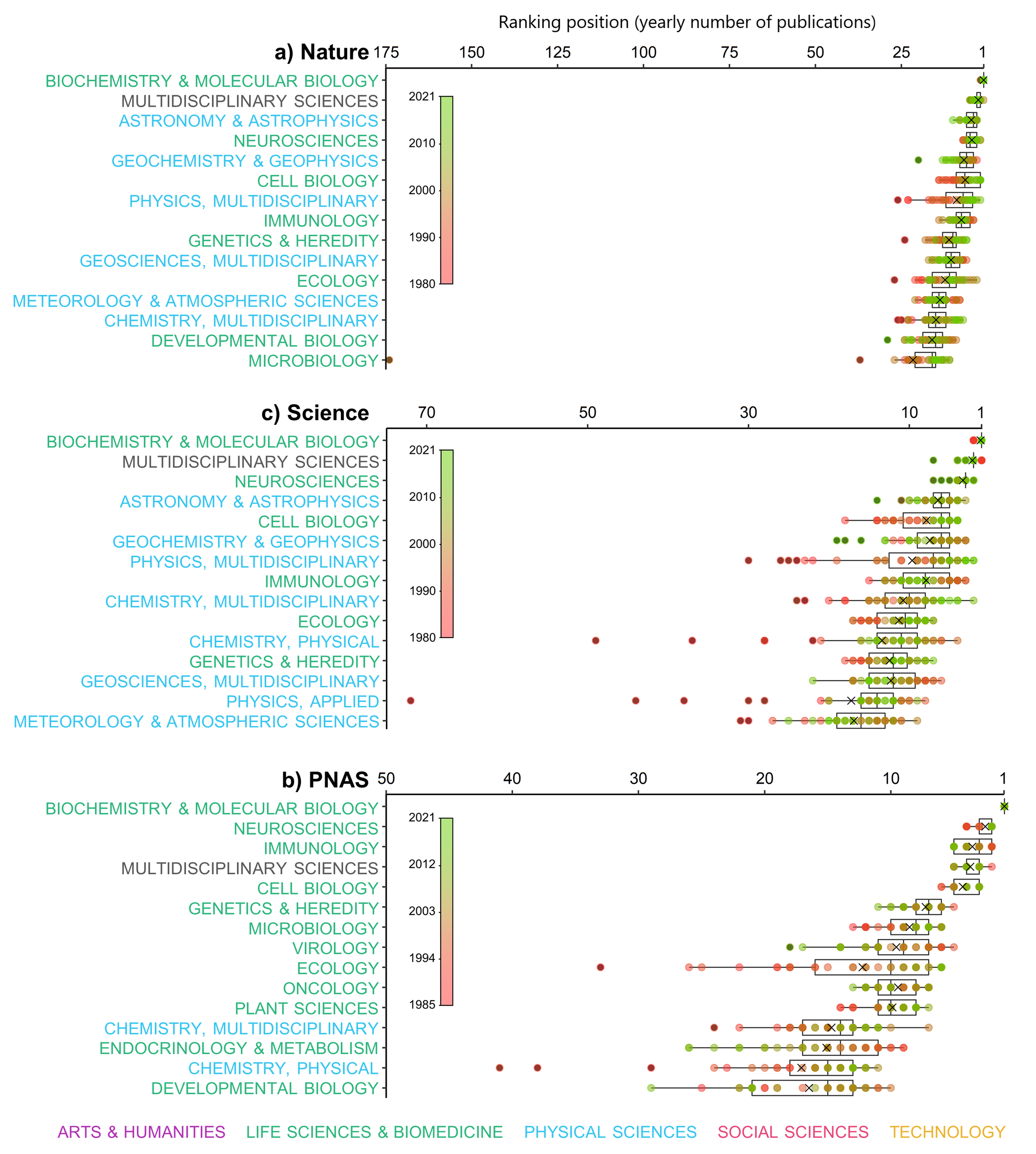

Supplement: S2 Fig — Position of each research area in the ranking of the number of publications in top multidisciplinary journals; a) Nature, b) Science, and c) PNAS; per year. Number one represents the research area with more publications. Only the 15 top-ranked research areas are shown, decreasingly sorted by the median. Research areas are classified by branches of knowledge and points are colored according to year. Note that the axes are represented at different scales. (TIF) [file pone.0314616.s002.tif]

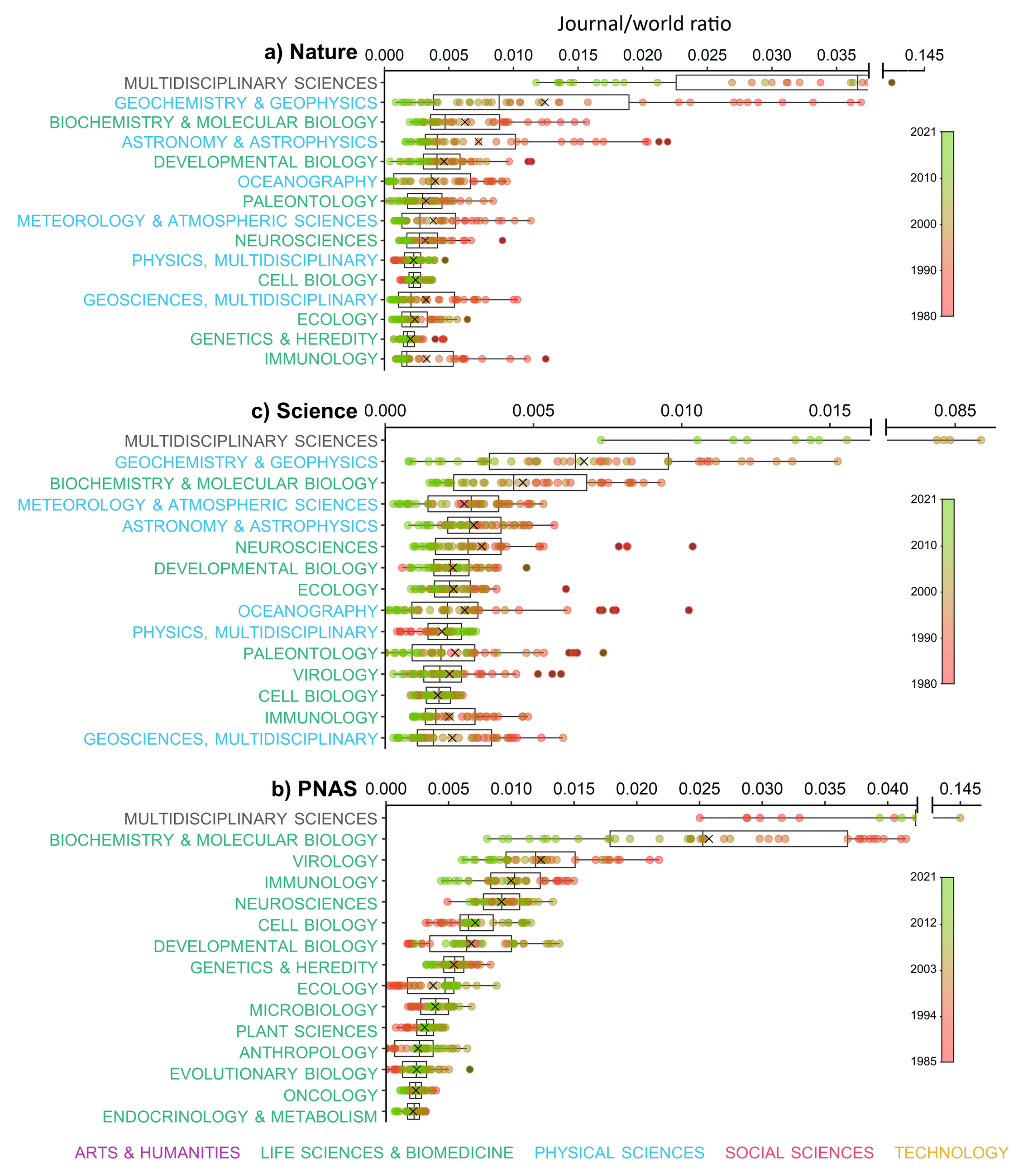

Supplement: S3 Fig — Ratio between the number of publications of each research area in top multidisciplinary journals; a) Nature, b) Science, and c) PNAS; and the total number of publications of that research area in the world (journal/world ratio). Only the 15 top-ranked research areas are shown, decreasingly sorted by the median. Research areas are classified by branches of knowledge and points are colored according to year. Note that the axes are represented at different scales. (TIF) [file pone.0314616.s003.tif]

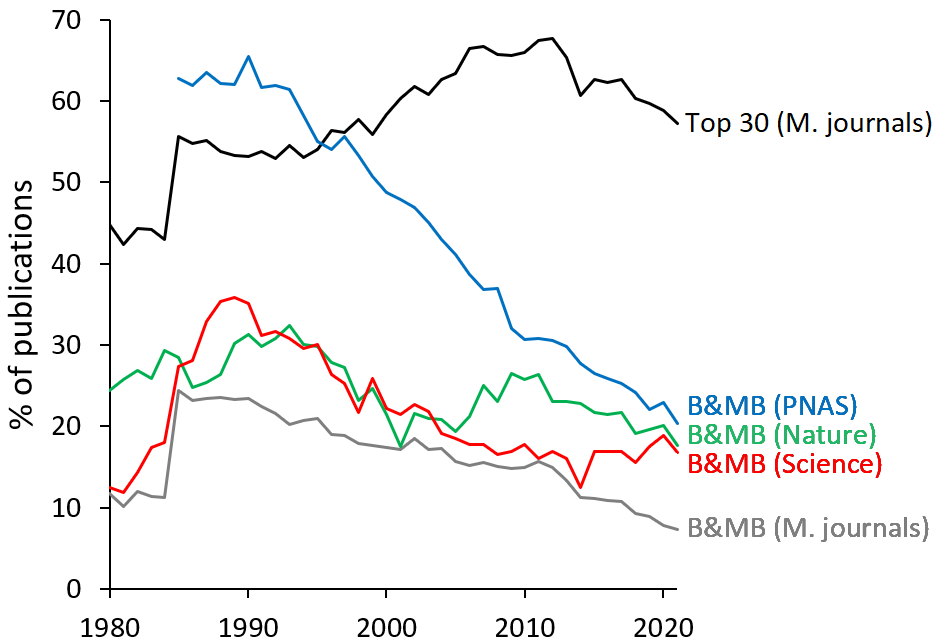

Supplement: S4 Fig — (TIF) [file pone.0314616.s004.tif]
