# Supplementary material for: A long-term assessment of the multidisciplinary degree of multidisciplinary journals
Source: PLoS One. 2024 Dec 2;19(12):e0314616. doi: 10.1371/journal.pone.0314616 (PMC11611101; doi:10.1371/journal.pone.0314616)
Supplement: S1 Table — The percentage of publications in each branch of knowledge in each decade is also shown. (DOCX) [file pone.0314616.s005.docx]

|  | 1980-1989 | 1990-1999 | 2000-2009 | 2010-2019 | 2020-2021 | Total |
| --- | --- | --- | --- | --- | --- | --- |
| **Number of multidisciplinary journals** | **50** | **71** | **90** | **108** | **93** | **127** |
| **Number of publications** | **79,988** | **112,999** | **124,530** | **522,383** | **143,346** | **983,246** |
| Life Sciences & Biomedicine | 37,871 (47.3%) | 51,083 (45.2%) | 66,448 (53.4%) | 359,553 (68.8%) | 89,623 (62.5%) | 604,578 (61.5%) |
| Physical Sciences | 12,782 (16%) | 21,767 (19.3%) | 24,928 (20%) | 69,739 (13.4%) | 23,413 (16.3%) | 152,629 (15.5%) |
| Multidisciplinary | 22,626 (28.3%) | 27,973 (24.8%) | 15,815 (12.7%) | 41,136 (7.9%) | 10,596 (7.4%) | 118,146 (12%) |
| Technology | 5,115 (6.4%) | 9,951 (8.8%) | 13,194 (10.6%) | 35,722 (6.8%) | 13,623 (9.5%) | 77,605 (7.9%) |
| Social Sciences | 1,273 (1.6%) | 1,679 (1.5%) | 2,491 (2%) | 13,299 (2.5%) | 5,422 (3.8%) | 24,164 (2.5%) |
| Arts & Humanities | 321 (0.4%) | 546 (0.5%) | 1,654 (1.3%) | 2,934 (0.6%) | 669 (0.5%) | 6,124 (0.6%) |
