# Supplementary material for: A long-term assessment of the multidisciplinary degree of multidisciplinary journals
Source: PLoS One. 2024 Dec 2;19(12):e0314616. doi: 10.1371/journal.pone.0314616 (PMC11611101; doi:10.1371/journal.pone.0314616)
Supplement: Attachment — Submitted filename: Response to reviewers.docx [file pone.0314616.s006.docx]

Reviewer #1: The manuscript has been much improved and is more moderated in its claims. However, in my view the response to my issues has been partial, and issues are still present though at a reduced level of severity.

****Our response:** Thank you for the time invested in this new round of revision. We hope the new version has fully resolved the issues raised.

There is still an issue of explaining how papers are classified into research areas.

****Our response:** To better contextualize and inform readers about how papers are classified into research areas, we have included a new subsection in the methodology. This subsection explains in detail how Web of Science and InCites classify journals and articles by Web of Science categories, and how the latter reassigns publications to other categories (lines 85-100). Additionally, we have improved the description of the data processing procedure based on InCites (lines 115-118).

The current text says this: “note that the research area of the publications is assigned by the Web of Science independently of the area of the journal in which they are published”

****Our response:** We have rewritten the sentence to clarify any doubts and provide more details about the classification system (lines 116-118).

The response to reviewers says this: “Our response: Web of Science assigns a research area to each paper, regardless of the area to which the journal belongs (this is true for journals of all areas, not only “multidisciplinary” journals). We have explained this in the new version of the ms (lines 96-97).” The text now clarifies that WoS classifies papers individually to areas and this is where the area classification of papers used comes from. However, the text does not explain how this classification is done. Some explanation of how this is done can be found here: https://incites.help.clarivate.com/Content/Indicators-Handbook/ih-document-reclassification.htm

****Our response:** Thank you for the suggestion. As mentioned above, we have included a new subsection with all those details (“Web of Science categories”) in the methodology (lines 85-100). Moreover, we have specified in the “Data gathering” section that the WoS algorithmically make the classification of publications for multidisciplinary journals according to cited references, when available (lines 115-118).

In Clarivate’s explanation are several important methodological points that the reader needs to understand in order to understand what is done in this paper. These are:
1 Clarivate assigns papers “using information found in the cited references of each publication” to make an algorithmic assignment.

****Our response:** We have included a new subsection with that information (“Web of Science categories”) in the methodology (lines 85-100). Moreover, we have mentioned the algorithmic reassignment in the “Data gathering” section (lines 115-118). In any case, regardless of the internal classification system, what we are addressing here is the outcome of the classification and its effects on different scales (see lines 421-443).

2 “In cases where it is not possible to accurately reassign publications (e.g., when the article does not have cited references), the articles are left as multidisciplinary.” This is important because it speaks to the meaning of the “multidisciplinary” category accounting for 12% of papers in these journals. In my first set of comments I thought this category might be important. If these are largely papers without references, this category is likely of little interest.

****Our response:** We have specified in the new subsection (lines 85-100) and in the “Data gathering” section (lines 115-118) that the reassignment is only made for some journals and when references are available. We are aware of this issue, but it is of little significance (not many publications do not have references) and does not affect our results and conclusions. Moreover, our approach is conservative, and the potential bias you mentioned only reinforces our message. If these unclassified papers were reclassified into other categories, the imbalances we are illustrating would likely be even more pronounced.

Also, the text talks about the difficulty of multidisciplinary papers finding a place to publish because in multidisciplinary journals they compete with molecular biology for space. The 12% of papers that are “multidisciplinary” seems to justify this point if we do not know what “multidisciplinary” actually means at the paper level. But if “multidisciplinary” at the paper level largely means papers with no references, then it is unclear if real multidisciplinary papers are even found in these journals at all.

****Our response:** We understand your reasoning. However, our statement about the difficulty of multidisciplinary papers finding a place to publish is not based on that percentage, but on the strong disbalance to highly cited categories. It is evident that if most multidisciplinary journals publish most of their articles in this “Biochemistry and Molecular Biology”, the space left for other types of articles, including multidisciplinary ones, is very limited.

3 In the response to reviewer the authors say WoS classifies all papers individually. Clarivate apparently does not do this. Instead, they say: “We apply reclassification to articles in the categories of Multidisciplinary Sciences and Medicine, General, and Internal”

****Our response:** We have specified in the new subsection (lines 85-100) and in the “Data gathering” section (lines 115-118) that the WoS algorithmically make the classification of publications for multidisciplinary journals according to cited references, when available.

Second point is to encourage more clarity in thinking about who is responsible for what. The paper seeks to go beyond description and make normative claims. That is, somebody is doing something wrong and should do better. This requires more work than is done here. Greater precision and clarity of thought would improve the paper and the conclusions drawn. Here are a few examples of what I mean. Lines 403-406 say: “Many science institutions and agencies utilize these categories for making rankings, journal evaluations, and even personnel selection within the academic career. Thus, the actual scope of a journal and its assigned category should be aligned accordingly” Use of passive voice in the second sentence conceals who is responsible. Presumably, Clarivate should align things. However, one could equally say that agencies and universities shouldn’t use this category. Or point out that places looking for Nature, Science or PNAS papers are essentially looking for molecular biologists and should understand that.

****Our response:** Here, we would like to remark that “thinking about who is responsible for what” is not our mission. We just aim to highlight that something is wrong and should be considered by the scientific community. Whether the effort in aligning the actual scope of a journal and its assigned category should be made by Clarivate, the agencies, and/or the journals is a matter of further discussion that goes beyond our study. We have added this sentence to be clear (lines 427-428).

Similarly, I said the importance of the WoS categories to authors is overblown in the paper. In the response the authors disagree, saying authors use them to figure out where to publish. The trouble is, this paper mostly focuses on Nature, Science and PNAS. And while it is true that when I write an information science paper, for example, I may look at the WoS categories to find a journal, nobody does that and decides to submit to Nature, Science and PNAS. Instead, they have a lifelong ambition to publish in Nature, Science or PNAS and think they may have at last produced something that could be published there. So while in general categories might have some use, they are of little use to authoris in relation to the journals focused on in this paper.

****Our response:** First, we do not believe that we are exaggerating the importance of WoS categories for authors (see line 421 for the only mention of authors in the Discussion). Second, in many scientific disciplines, such as the exact sciences, WoS categories can indeed be relevant for authors, who are continuously evaluated (for contracts, projects, etc.) based on metrics such as the number/proportion of papers published in the first quartile of their categories. Third, please note that, while we discuss the cases of *Nature*, *Science*, and *PNAS* in detail, the article does not focus on them exclusively. This is actually one of the strengths of our study. We simply found it appropriate to dedicate a specific section to them, as they are unique and significant within this ecosystem, as you rightly point out.

On the DORA point, line 417, DORA was about research assessment, not about scope of journals. A journal seeking to publish high impact research therefore does not contravene DORA because the journal is not performing research assessment, as is required to be relevant to DORA.

****Our response:** Certainly, the issue does not lie with the journal itself. We have clarified this point in the text (lines 437-443).

Line 420, on planetary relevance, “the attention devoted to the different research branches and areas in multidisciplinary journals meets the demands of society and the planet is highly questionable. . .” I suggested this statement was not supported by the analyses. The response points to the final words about doing more research to say it is fine. I re-emphasis here the judgement – “questionable” – and that nothing in the analysis presented supports that point. Of course, life, the universe and everything deserves further research, but that still doesn’t justify making unsupported claims.

****Our response:** Given that this sentence appears to cause confusion and does not contribute any significant ideas to the paper, we have removed it.

Reviewer #2: My major questions/concerns are still not addressed. Their response to my request to provide more details so that the reader could reconstruct the study: "our methodological procedures are totally replicable by any other researchers...The other classifications come from the Web of Science, the Journal Citation Reports, and the InCites Dataset. As these databases are well-known by scientific readership, we believe that it is not necessary to explain details about their functioning" is not satisfactory. Despite having extensive experience in this area I would not be able to reproduce the study. Also given that the main topic of their paper deals with classification they need to explain the classification(s) used by Clarivate.

****Our response:** Thank you once more for the time invested in reviewing our manuscript. This issue is also shared by Reviewer 1; following the suggestions of both reviewers, we have introduced an entire subsection to better describe all these aspects concerning the Web of Science categories (lines 85-100). Moreover, we have provided more details about the classification system in the “Data gathering” section (lines 115-118). In any case, please note that a better understanding of how WoS and InCites work does not affect reproducibility of our study, as any researcher who follows the procedures described in the “Data gathering” and “Data treatment and analyses” sections should obtain the same dataset and results as we did.

First, the authors should specify after they obtained ISSNs using the Journal Citation Reports what database they used (Web of Science or InCites Dataset) to collect data on publications. They should then specify what fields they used. My best guess is that they used: PY (Year Published), SO (Publication Titles), SU (Research Area) and, WC (Web of Science Categories) fields. However, they should specify this.

****Our response:** We have clarified issues related to data extraction, indicating that the data are extracted from InCites and not from Web of Science (lines 111-115).

They should also then say something about Web of Science categories - how were they constructed, how many are there, how they address the scenario of multiple subject categories being assigned to a journal.

****Our response:** As mentioned above, we have specified in the new subsection (lines 85-100) and in the “Data gathering” section (lines 115-118) all those details.

They also say in the methods that they included in the data set all the journals that have at least one of their editions in the period between 1997 and 2020 classified as "Multidisciplinary Sciences." What are the implications? How many such journals are there? Is there a difference between journals being classified in this category throughout the period of the study and the ones that were classified only occasionally? Also, are there journals that have multiple Web of Science categories assigned to them? If so, how many?

****Our response:** Of the 127 journals considered in our study, 55 (43%) were included in the category “Multidisciplinary Sciences” for at least half of the study period, and only 10 (8%) were included for <5 years. Thus, very few journals were classified occasionally as multidisciplinary. Also, during the study period, 86 journals (68%) were exclusively categorized as “Multidisciplinary Sciences”. Therefore, we can say that our selection of journals has greatly contributed to multidisciplinary sciences during our study period. For clarity, we have included these data in the new version of the ms (lines 107-111).

The authors should also provide more details about the field SU (Research Area). How many Research Areas are there in Web of Science?

****Our response:** There are 254 research areas, as we already specified in the previous versions of our ms (line 89 in the current version).

How are they assigned?

****Our response:** Articles inherit the categories of the journal in which they are published, with some exceptions, as we now specify in the new subsection on “Web of Science categories” (lines 85-100).

Does a paper have multiple Research Areas? How is such a case resolved in this study?

****Our response:** Please note that this was already specified in the previous version of our ms (lines 135-139 in the current version): “Publications included in more than one research area were considered in each of the areas they were included (but note that only 3.1% of publications were included in more than one area, and that these multiple areas were often closely related, e.g., “Computer Science, Hardware & Architecture” and “Computer Science, Theory & Methods”).”

To avoid confusion maybe the authors should use different terminology when they talk about classes for journals and papers.

****Our response:** To avoid confusion, in the new version we clarify this distinction by using "category" for the areas of the journals and "research areas" for the areas of the publications.

Finally, the authors should provide tables that list "branches of knowledge" and Web of Science categories assigned to each.

****Our response:** As specified in the text (lines 119-122), we base this classification on a previously published article where this table can be found (Arroyo-Machado & Torres-Salinas, 2021). Therefore, we do not believe it is necessary to include the same table in our article.

These clarifications are essential before the findings of the study can be properly assessed.

****Our response:** We hope that our responses and the improvements made in the new version have fully resolved the issues raised.

A few additional comments:

In lines 52-53 the authors say: "multidisciplinary journals usually tend to accept articles based on their potential citability (4,5)". I don't think that these references provide claims that the criterion for the acceptance of papers by journals is the potential citability of articles. This is a very strong claim which is not founded in the supported evidence.

****Our response:** In this case, we disagree with you. The references provided do fully support the message we convey. Reference 4 states: “Existing multidisciplinary journals publish selectively most-cited papers from fields with high citation density”; and reference 5 states: “Articles in them [multidisciplinary journals] are explicitly chosen not only because they move their specific literatures in important ways, but also because of their potential to interest–and to influence–multiple segments of the scientific community” and “The articles appearing in them are not inherently of greater interest […] but, in keeping with differences in JIF, they can attract a markedly greater number of out-of-field citations”.

In lines 63-64 the authors say: "the growing competitiveness of the increasingly crowded multidisciplinary discipline may jeopardize its multidisciplinary nature." What is "multidisciplinary discipline"?

****Our response:** We have specified that we refer to the multidisciplinary category (line 61).

In line 82 the authors say: "the proportion of publications in the journals that belong to the top area". How do they define top area (the one with the largest number of articles or the one with most citations)?

****Our response:** The one with the largest number of articles; we have now specified it in the text (line 79).

In lines 226-232 the authors say "Number one represents the research area with more publications." What is "number one" what is "the research area with more publications"?

****Our response:** We have made some changes in the Figure 2 (including 1 instead of 0 in the y axis of the panel a) and its legend (lines 242-249) to make them clearer.

Also why the x axis of Figure 2b goes from 1 to 8000 and Figure 2c goes from 150 to 1. I know understand that the scales are different, but the axes should be consistent (either from smallest to the largest or from the largest to the smallest number).

****Our response:** The axes are different because they represent different variables (number of plublications in the case of panel b and ranking position in the case of panel c). Additionally, they are inverted relative to each other (as we now indicate in the figure legend) to make them more easily interpretable and comparable.

As it comes to Figure 2a I am not sure what the major take-away message is, nor what do bars represent.

****Our response:** This panel shows the interannual variation in the rankings of each multidisciplinary journal, with the main message being that this variation is generally lower for journals in the top categories compared to all other journals. This idea is mentioned in lines 258-259. Then, details on the top categories are provided in Figure 2c.

The authors have still not labeled x-axes for Figure 3a and 3b, so I don't know what number 0, 5, 10 and 0, 1, 2, 3, 4, 5, 6, 7, 8 represent.

****Our response:** We are sorry; in the new version, we have indicated in the figure that the x-axes represent the percentage of total publications in the world that belongs to each research area.

There is no reason for Figure 3a to have different units than the one on 3b. Also as far as I can see in both 3a and 3b there is no area that has value above 5, so why does one go to 10 and another to 8? Both should use 0, 1, 2, 3, 4, 5, 6.

****Our response:** First, given that Figure 3b is a zoom-in of Figure 3a, units must necessarily be different. Second, the cropping done to each graph is simply for better visualization (of not only the points, but also the names of the research area).

Finally, the authors claim that: "All data are available from the InCites dataset (https://incites.clarivate.com/)" and the data is fully available without restriction. However, when I followed the link I was asked for the subscription.

****Our response:** It is true that an institutional account is required to access the data, so we have included the main dataset used in our analysis (https://doi.org/10.5281/zenodo.13269363).
