# Supplementary material for: A long-term assessment of the multidisciplinary degree of multidisciplinary journals
Source: PLoS One. 2024 Dec 2;19(12):e0314616. doi: 10.1371/journal.pone.0314616 (PMC11611101; doi:10.1371/journal.pone.0314616)
Supplement: Attachment — Submitted filename: Response to reviewers.docx [file pone.0314616.s007.docx]

*Please note that the line numbers referenced correspond to the updated version of the manuscript with track changes.*

**Reviewer #1:**

The analysis shows that life sciences is over represented in these journals compared to all of science. This is well known. The analysis also shows that this may be decreasing, which may be less well known. The methods are now better explained and the conclusions somewhat moderated.

****Our response:** Thank you for the time dedicated to this new round of revision. We appreciate your acknowledgment of one of the values of the study, namely the temporal component, and the improvements made to clarify the methods and moderate the discussion. As you will see below, we have made an additional effort to accommodate the Discussion to your criticisms.

This paper wants to conclude that Science, Nature and PNAS shouldn’t be classified as multidisciplinary because 13% of their papers are in biochemistry & molecular biology, and that is the largest WoS category in these journals. Even at the disciplinary level shown in figure 1, more than life sciences is published in these journals. How are they supposed to be classified? Given the data in the paper, it is easy to conclude that multidisciplinary is indeed an accurate classification of these journals. Therefore, I disagree with the conclusions of the paper. There is no problem here, not for the journals, not for Clarivate, not for scientists nor for the institutions that employ them.

****Our response:** We appreciate your emphasis on these comments, as they prompted us to reconsider our conclusions. Consequently, we have removed the part suggesting that organizations responsible for classifying and rating scientific journals should assess whether the label ‘Multidisciplinary’ accurately represents the scope of *Nature*, *Science*, *PNAS*, and other journals categorized as multidisciplinary. Instead, in the revised version, we now state that “the multidisciplinary label need not be removed from these journals” (lines 434-435), and we shift the focus to researchers and scientific agencies, encouraging them to be aware of the existing polarization of multidisciplinary journals toward specific areas (lines 477-479; we have also included this idea at the end of the Abstract, lines 40-42). Additionally, we have briefly expanded the discussion on issues arising from this bias to stimulate further debate and research on this topic (lines 427-475). Thus, we hope this revised version of the Discussion contributes to promoting more balanced publication opportunities and fairer research evaluations, rather than critiquing the editorial decisions of these journals or Clarivate’s classification efforts.

**Reviewer #3:**

The manuscript presents an interesting study of the multidisciplinarity degree of multidisciplinary journals and changes in that degree over time. I see that the first round of reviewers' comments contain some concerns and some divided opinions, hence I've tried to give this a careful consideration.

****Our response:** Thank you for your time and consideration. We hope that the changes made in the revised version satisfactorily address your concerns.

I believe the methodology used has been appropriate and the results support the conclusions, but perhaps some of the conclusions, or the concerns described from line 400 onward, need somewhat more explanations and discussion.

****Our response:** We appreciate your recognition that the methods are appropriate and the results support the main conclusions. We have made some changes to the final section of the Discussion (especially, lines 427-498) that we hope have clarified your concerns (please see below for details).

I agree with the first concern, that these categories are used for many reasons and thus it would be important that they align with the journals accordingly. A mismatch between journals and their categories would lead to inaccurate rankings and evaluations. But is this a problem that the journals should try to solve or Clarivate who has assigned the categories? What level of multidisciplinarity is needed for a journal to be appropriately called multidisciplinary? And on the other hand, what level of specialization is needed for a journal to move from multidisciplinary category to a subject specific category? How many articles in Biochemistry and molecular science should Nature have for it to belong to that category?

This leads to a more fundamental question of the reasons behind the degree of multidisciplinarity and connects to the second concern that the authors have stated. How much of the shown specialization of multidisciplinary journals is an intentional editorial choice by a journal? Is it the journal, editor, or reviewers that have influenced the direction the journals take? Does the degree of multidisciplinarity change when the editor changes? Is it an intentional decision that certain articles will not be published, making it difficult for more multidisciplinary articles to be published? This goes to the reasons for the changes in the degree of multidisciplinarity and I understand that this was beyond the scope of this study, but it would be interesting to read how the authors see these issues o concerns.

The third concern raised by the authors (line 415 onward) has two parts that warrant some more thought. First, is it an intentional effort, a choice, that the journals focus on more highly cited fields? Is it an editorial decision to boost the IF? Or could it be that the more prominent field aligns better with the editor or current reviewers? Again, there are probably no clear answers.

****Our response:** The questions you propose are indeed thought-provoking, so we have included several of them as open questions in the new version of the Discussion to encourage debate and inspire future research on this topic (lines 451-457, 472-475). As you rightly note, there may not be definitive answers here, or at least our data do not allow us to fully resolve these questions. It does seem reasonable to suggest that this polarization may serve as a strategy to increase the impact factor. However, while this aligns with our findings, we prefer not to speculate further, leaving the door open for future debate and research (lines 451-457).

But what I do think needs a bit more explanation, although most readers probably have some idea about what DORA is, is why this would be against the essence of DORA? I'd rather see this written out than leave it for the reader to contemplate. Besides, I'd very much like to read about the authors thinking on this matter.

****Our response:** Following your suggestion, we have revised this paragraph (lines 459-475). We hope the result has addressed the issues raised.

And finally the fourth concern, whether the journals meet the demands of the society and the planet. I believe this is too vague and too ideological of a statement. While this might be true, this is not something that I see evidence of in the results (as mentioning the society and the planet takes the argument to an ideological level, rather than factual).

****Our response:** We agree with you, so we have removed that sentence.

Regarding the categorization and the journals choice to publish what they want (that fits their scope and mission statements), I fully agree that journals may shift their focus how they please and it would be the job of Clarivate and other indexes to reassign the journals if and when appropriate. But I'd like to add that readers that are not fully knowledgeable about how journals are assigned to categories, most likely expect multidisciplinary journals to be truly multidisciplinary, ie not be biased to published more articles from specific fields. Even for this reason, I think this study makes some valuable contributions.

****Our response:** We fully agree with your reflections. We have considered them in the new sentences at the end of the Abstract (lines 40-42) and in the final paragraph of the Discussion (lines 477-479).

My final point is about the choice of wording at some places. The authors write for instance "PNAS have consistently increased its degree of multidisciplinarity" (line 396). This goes back to my earlier point, whether it is a intentional effort to increase this degree or some unintentional effect of for instance the background of the editor or the reviewers. The wording that the authors have used suggests that this would be intentional, but I'm not sure if that can be read from the results. On the other hand, "the degree of multidisciplinarity in PNAS has consistently increased" would not suggest anything, it would only state that this is the case.

****Our response:** We appreciate the suggestion. The proposed sentence conveys exactly the meaning we intend, so we have changed our original sentence accordingly (lines 423-424). Moreover, we have revised several parts of the manuscript, with special emphasis on the Abstract and the first paragraph of the Discussion, to enhance clarity.
